# Free Space Ground to Satellite Optical Communications Using Kramers–Kronig Transceiver in the Presence of Atmospheric Turbulence

**DOI:** 10.3390/s22093435

**Published:** 2022-04-30

**Authors:** Mahdi Naghshvarianjahromi, Shiva Kumar, M. Jamal Deen

**Affiliations:** Department of Electrical and Computer Engineering, McMaster University, Hamilton, ON L8S 4K1, Canada; skumar@mcmaster.ca (S.K.); jamal@mcmaster.ca (M.J.D.)

**Keywords:** geosynchronous equatorial orbit (GEO) satellite to GEO satellite (sat-to-sat), GEO satellite to ground station (downlink), ground station to GEO satellite (uplink), atmospheric turbulence, free space communications (FSO), Krammer–Kronig (KK) transceiver, coherent transceiver, optical wireless communications (OWC)

## Abstract

Coherent detection provides the optimum performance for free space optical (FSO) communication systems. However, such detection systems are expensive and require digital phase noise compensation. In this paper, the transmission performance of long-haul FSO system for ground-to-satellite communication based on a Kramers–Kronig (KK) transceiver is evaluated. KK transceivers utilize inexpensive direct detection receivers and the signal phase is retrieved from the received current using the well-known KK relations. KK transceivers are not sensitive to the laser phase noise and, hence, inexpensive lasers with large linewidths can be used at the transmitter. The transmission performance of coherent and KK transceivers is compared in various scenarios such as satellite-to-ground, satellite-to-satellite, and ground-to-satellite for weak, moderate, and strong turbulence. The results show that the transmission performance of a system based on the KK transceiver is comparable to that based on a coherent transceiver, but at a significantly lower system cost and complexity. It is shown that in the absence of turbulence, the coherent receiver has a ~3 dB performance advantage over the KK receiver. However, in the presence of strong turbulence, this performance advantage becomes negligible.

## 1. Introduction

Typical modulation formats for free space optical (FSO) communications, such as satellite-to-ground communications, are on–off keying (OOK), differential phase-shift keying (DPSK), and polarization shift keying (POLSK) that use a direct detection (DD) receiver, or binary phase-shift keying (BPSK) or quadrature phase-shift keying (QPSK) using a coherent receiver [1,2,3,4]. Although the OOK, DPSK and POLSK modulations with DD receivers are inexpensive, the bit/symbol efficiencies of these modulation techniques are quite low. This problem is solved using coherent detection in which both amplitude and phase of the optical signal can be extracted [5,6]. Among various detection approaches, coherent detection provides the best sensitivity and high spectral efficiency. However, coherent receivers are expensive, they use four photodetectors (PDs) per polarization and they require a laser which acts as the local oscillator (LO). In contrast, DD receivers require only one PD and do not need a LO. Besides, for phase-modulated signals, laser phase noise is a serious problem and, therefore, expensive external cavity lasers (ECL) with very small linewidths should be used at both the transmitter and at the receiver for coherent detection systems.

In this paper, we propose the Kramers–Kronig (KK) transceiver for a long-haul FSO communication scheme in the presence of turbulence. KK receivers make use of inexpensive DD receivers. The signal phase can be extracted from the photocurrent of the PD using a Hilbert transform in digital domain. Therefore, phase-modulated signals, such as quadrature amplitude modulation (QAM), can be detected using KK relations even though a DD receiver is used. Unlike coherent receivers, a KK receiver does not require a laser at the receiver and a communication system based on a KK transceiver is not sensitive to the transmitter laser phase noise due to the use of direct detection. Therefore, inexpensive lasers with larger linewidths can be used at the transmitter. Also, digital phase noise compensation techniques are not required for the FSO system based on KK transceivers. The transmitter of a KK transceiver consists of a single Mach–Zehnder modulator (MZM) since the driving voltage is real and positive while that of a coherent transceiver requires a more complex optical in-phase and quadrature (I-Q) modulator consisting of two MZMs. Thus, KK transceivers enable high spectral efficiency communications at a lower cost. However, these benefits come with a cost—to retrieve the signal phase at the receiver, we need to add a direct current (DC) component to the signal at the transmitter which lowers the power efficiency of the transmitter, just as in intensity modulated direct detection (IMDD) systems.

**Figure 1:**
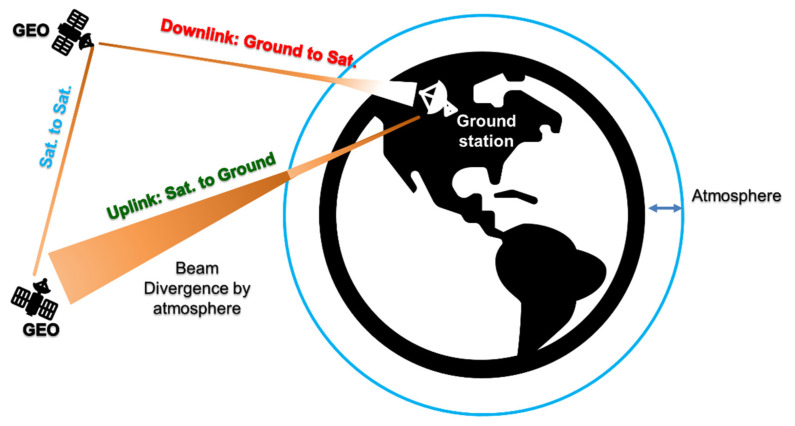
Free space optical (FSO) communications scenarios between geosynchronous equatorial orbit (GEO) satellites and ground station.

The KK relations are widely used in different areas of physics and engineering [7,8,9,10,11]. In [12,13,14], KK relations are used to design the DD coherent receivers for fiber optic communications which makes use of minimum phase signals. Recently, KK receivers have been experimentally implemented for FSO systems for short ranges of 55 m [15] and 20 m [16]. At these short ranges, the effect of atmospheric turbulence is negligible. However, to our knowledge, the performance of KK receivers in long-haul FSO communication systems in the presence of atmospheric turbulence has not been evaluated. In this paper, we study KK transceivers for FSO systems with QAM-16, 40 Gb/s data rate, and 35,000 km reach for three different scenarios: (i) Geosynchronous equatorial orbit (GEO) satellite to GEO satellite (sat-to-sat); (ii) GEO satellite to ground station (downlink); and (iii) ground station to GEO satellite (uplink), as shown in Figure 1. Besides fading, atmospheric turbulence creates extra laser beam divergence in uplink and downlink (see Figure 1) that can lead to extra path loss. We compared the proposed FSO KK scheme with a FSO coherent transceiver for downlink and uplink scenarios over weak, moderate, and strong atmospheric turbulence. The results show that KK transceiver can be used efficiently with performance comparable to a coherent transceiver for space communications.

The KK receivers are compatible with multi-channel systems. If the bandwidth of the transmitted signal is less than or equal to the bandwidth of the PD (for example, orthogonal frequency division multiplexing (OFDM)), then the KK receiver extracts the phase, and the complex signal is demultiplexed in digital domain (in the case of OFDM, FFT is used for demultiplexing). If the bandwidth of the transmitted signal exceeds the bandwidth of a PD (for example, wavelength division multiplexing (WDM)), then the received signal is demultiplexed using wavelength division demultiplexer and the demultiplexed signals pass through their respective PDs and KK receivers.

## 2. Background on Atmospheric Turbulence for FSO System

In a FSO system, the channel capacity is limited by atmospheric turbulence. Turbulence leads to random variations in refractive index which causes random changes in the amplitude and phase of the received optical signal. The fluctuation of the received signal intensity resulting from turbulence leads to performance degradation and limits the capacity. A statistical model for the optical intensity fluctuation at the receiver due to the atmospheric turbulence was derived in ref. [17] and the beam width is maximized to optimize the channel capacity. The impact of turbulence on the bit error rate (BER) was considered in refs. [18,19].

One of the major challenges for using the coherent detection in FSO communication systems in the presence of atmospheric turbulence is the poor mode mixing efficiency [20,21,22]. The spatial part of the optical field in the presence of atmospheric turbulence can be expressed as the superposition of Laguerre–Gaussian (LG) modes. Even if the optical field at the transmitter is Gaussian (i.e., LG_00_ mode), after propagating through the turbulent medium, its energy is transferred to higher order LG modes due to the random refractive index of the turbulent medium. Typically, the spatial part of the local oscillator (LO) of the coherent receiver is Gaussian and the higher order modes of the received field do not efficiently mix with the Gaussian field of the LO due to mode orthogonality. Hence, data-LO mixing efficiency can be degraded by >20 dB due to mode mismatch between the LO and data beams [20,21,22]. However, an IMDD FSO link with free-space coupled photo-detector (PD) is not significantly affected by turbulence-induced modal coupling if the receiver aperture can collect most of the received field since the IMDD system does not require LO. Ref. [20] proposed a pilot assisted self-coherent detection to solve the mode mismatch problem. In this scheme [20], a pilot beam is transmitted along with the data beam from the transmitter. The pilot and data beams are more likely to undergo the similar mode-coupling to higher order LG modes due to atmospheric turbulence. The received pilot beam replaces the LO of coherent detection. However, mode mixing efficiency of the self-coherent detection is much higher than that of coherent detection. Experimental results of ref. [20] shows an average mixing loss of ~3.3 dB for self-coherent detection. The proposed KK scheme for FSO in the presence of turbulence has some similarities and differences with self-coherent detection: (i) both schemes use a pilot beam—the pilot beam of the self-coherent scheme has a frequency offset from the data beam whereas the pilot beam of the KK scheme is in-band; (ii) in KK scheme, the phase is extracted using the Hilbert transform whereas in self-coherent detection, the beating between the pilot and data is used for the data retrieval; (iii) mode mixing efficiency of the KK scheme is similar or slightly higher than that of self-coherent detection since the pilot is in-band in the KK scheme; and (iv) both schemes suffer from poor power efficiency as compared to coherent detection since a fraction of the transmitter power is wasted in sending the pilot signal.

## 3. KK-Based FSO Communications

**Figure 2:**
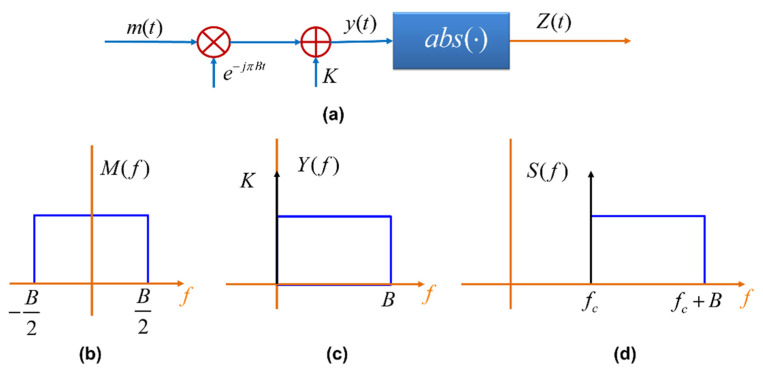
(**a**) Schematic of the KK digital transmitter, (**b**) message spectrum, (**c**) spectrum of *y(t)*, and (**d**) spectrum of optical transmitter output, i.e., after optical modulation.

In a KK-based FSO receiver, because a direct detection receiver is used, the optical signal phase is lost. However, it is possible to retrieve the signal phase from the KK relation. Let m(t) be a complex message signal whose spectrum lies between −B/2 and B/2 (see Figure 2b), where B/2 is the electrical carrier frequency. The carrier is modulated by m(t) and a DC bias *K* is added (see Figure 2a). Let

(1) $$y(t)=K+m(t)e^{-j\pi Bt}$$

The absolute of *y*(*t*), *Z*(*t*) is the input of a dual drive Mach-Zehnder modulator. Let the complex field output of the optical transmitter be [6]

(2) $$s(t)=\sqrt{P_{0}}Z(t)e^{-j(2\pi f_{c}t+\theta (t))},$$

where $f_{c}$ is the optical carrier frequency, θ(t) is the laser phase noise at the transmitter and $P_{0}$ is the carrier power. The DC bias, *K* leads to a pilot beam as shown in Figure 2d. It can be shown that y(t) is a minimum phase signal if and only if the winding number of the time trajectory in the complex plane is zero [12,13,14]. The condition |*K*| > max [|*m*(*t*)|] is sufficient for guaranteeing the minimum phase properties [14]. Let

(3) $$y(t)=Z(t)e^{j\varphi (t)},$$

where Z(t) and φ(t) are the amplitude and phase of y(t). When y(t) is a minimum phase signal, Z(t) and φ(t) are related by the Hilbert transform,

(4) $$\varphi (t)=\frac{1}{\pi }p.v.\int_{-\infty }^{\infty }dt^{\prime }\frac{\log[Z(t^{\prime })]}{t-t^{\prime }},$$

where *p.v.* stands for principal value.

**Figure 3:**
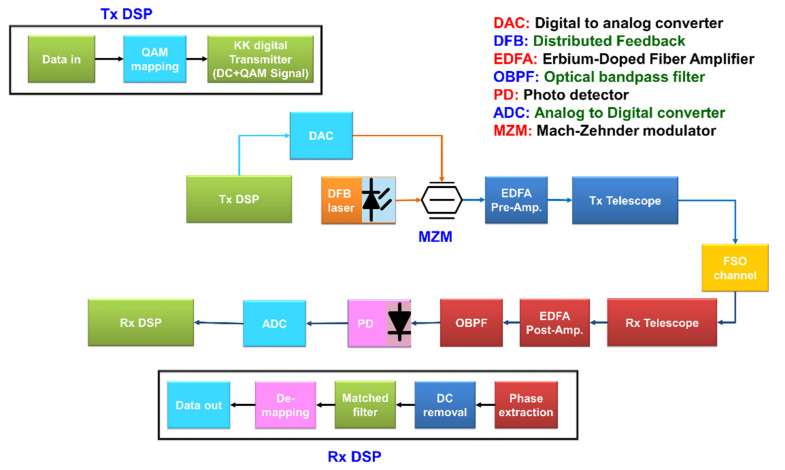
Schematic of the KK transceiver for FSO communications.

Figure 3 shows the FSO system based on KK relation. At the receiver, a direct detection receiver is employed. The photocurrent is given by

(5) $$I(t)=Rr(t)=RH|s(t){|}^{2},$$

where *R* is the responsivity of the photodiode, *r*(*t*) is the received power, and *H* is the channel gain. Using Equation (2) in Equation (5), we see that the photocurrent does not depend on the transmitter laser phase noise, θ(t). In Equation (5), noise is neglected. Using the Equations (2) and (3) in Equation (5), we find

(6) $$Z(t)=\sqrt{\frac{I(t)}{RHP_{0}}}.$$

Using Equation (6) in Equation (4), we obtain

(7) $$\varphi (t)=\frac{1}{2\pi }p.v.\int_{-\infty }^{\infty }dt^{\prime }\frac{\log\left[I(t\prime )/(RHP_{0})\right]}{t-t^{\prime }},$$

Thus, we can retrieve the phase of y(t). From Equations (1), (3) and (6), we have

(8) $$m(t)=\left[\sqrt{\frac{I(t)}{RHP_{0}}}e^{j\varphi (t)}-K\right]e^{j\pi Bt}.$$

The convolution in Equation (7) can be conveniently implemented in frequency domain as

(9) $$\tilde{\varphi }(\omega )=\frac{i}{2}sign(\omega )F\left\{\log\left[\frac{I(t)}{RHP_{0}}\right]\right\},$$

(10) $$\varphi (t)=F^{-1}\left\{\tilde{\varphi }(\omega )\right\},$$

where *F* denotes the Fourier transform and sign(⋅) is the sign function,

(11) $$sign(\omega )=\{\begin{array}{ccc} -1 & for & \omega >0\\ 0 & for & \omega =0\\ 1 & for & \omega <0 \end{array}$$

As shown in Figure 3, the receiver photocurrent passes through an analog-to-digital converter (ADC) and then through the receiver digital signal processing (DSP) unit (Rx-DSP). The phase retriever (Equations (9) and (10)) is implemented in the Rx-DSP. The complex message signal m(t) is estimated using Equation (8). The logarithm appearing in Equation (7) introduces spectral broadening and hence, digital up-sampling of the received photocurrent is required. The block diagram of KK transceiver can be more generalized for wavelength division multiplexing (WDM) by adding a multiplexer and a demultiplexer at the transmitter and receiver, respectively.

## 4. FSO System Design

As shown in Figure 3, the output of MZM passes through a pre-amplifier with gain *G*_1_ and the received signal passes through a post-amplifier with gain *G*_2_. Let *P_in_* and *P_out_* be the outputs of the MZM and post-amplifier, respectively. They are related by

(12) $$P_{out}=G_{1}AG_{2}P_{in},$$

where *A* is the path loss due to FSO link. As shown in Figure 1, *A* is different for three different scenarios: (i) satellite-to-satellite (sat-to-sat), (ii) satellite-to-ground (downlink), and (iii) ground-to-satellite (uplink) communications. In this paper, we assumed that a Gaussian laser beam is used at the transmitter. The path loss for the sat-to-sat communications is [23,24,25,26]:

(13) $$A_{Sat.-Sat.}=\frac{D_{T}^{2}D_{R}^{2}}{L^{2}\lambda^{2}}\eta_{T}T_{T}(1-L_{P})T_{R}\eta_{R},$$

where *L* is the distance between transmitter and receiver, λ is the wavelength, and *D_T_* and *D_R_* are the lens diameters of transmitter and receiver telescopes, respectively. *T*_T_ and *T*_R_ are the transmission factors (≤1) of the telescopes. $\eta_{T}$ and $\eta_{R}$ are the transmitter and receiver efficiency, respectively. *L_P_* is the pointing loss due to misalignment of transmitter and receiver. The path loss of downlink is [23,24,25,26]

(14) $$A_{downlink}=A_{Sat.-Sat.}\times 10^{-\frac{A_{atm}}{10}},$$

where *A_atm_* is the attenuation of the atmosphere in [dB]. The beam divergence due to the atmosphere for the downlink is ignored as it is negligible [21]. For the uplink, the attenuation due to turbulence and beam divergence is [20,21,22,23],

(15) $$A_{uplink}=\frac{D_{R}^{2}}{L^{2}\left(\theta_{T}^{2}+\theta_{atm}^{2}\right)}\eta_{T}T_{T}(1-L_{P})T_{R}\eta_{R}\times 10^{-\frac{A_{atm}}{10}},$$

where the divergence angle due to telescope at the transmitter is [23,25]

(16) $$\theta_{T}=\frac{\lambda }{D_{T}},$$

and additional divergence due to turbulence is

(17) $$\theta_{atm}=\frac{\lambda }{r_{0}},$$

where *r*_0_ is the *Fried parameter* [23].

Let *NF*_1_ and *NF*_2_ be the noise figures of the pre- and post-amplifiers, respectively. The effective noise figure is [6]

(18) $$NF_{eq}=NF_{1}+\frac{NF_{2}}{G_{1}A}-\frac{1}{G_{1}}.$$

Typically, $G_{1}>>1$ and the last term in Equation (18) can be ignored. From Equation (18), we see that if $G_{1}A<<1$ (which is typically the case), then the effective the noise figure is mainly determined by $NF_{2}$ (i.e., first term on the right side of Equation (18) is also negligible compared to the second term) and, hence, an amplifier with relatively low noise figure should be used as the post-amplifier.

Assuming that the noise of the optical amplifier and background noise to be white, then the noise power spectral density per polarization is

(19) $$\rho =\rho_{ASE,eq}+\rho_{bg},$$

where $\rho_{bg}$ is the power spectral density (PSD) of background radiation noise and $\rho_{ASE,eq}$ is the equivalent PSD of the amplified spontaneous emission (ASE) noise given by [6]

(20) $$\rho_{ASE,eq}=\left(G_{eq}NF_{eq}-1\right)\frac{hf}{2},$$

(21) $$G_{eq}=G_{1}AG_{2},$$

where *h* is Planck’s constant, and *f* = *c*/*λ* where *c* is the speed of light. The main source of background radiation is the sun, and its PSD [3,4] is,

(22) $$\rho_{bg}=m\frac{\lambda_{s}^{4}}{c}R(\lambda_{s}),$$

with

(23) $$R(\lambda_{s})=\frac{2hc^{2}}{\lambda_{s}^{5}}\frac{1}{\exp(hc/\lambda_{s}kT_{se})-1},$$

and

(24) $$m=A_{eff}\frac{D_{R}^{2}\pi }{4L_{s}^{2}\lambda_{s}^{2}},$$

where $A_{eff}$ is the area of source that can be seen by lens of receiver telescope, *k* is Boltzmann’s constant, $\lambda_{s}$ is the background radiation source wavelength at temperature *T_se_*, and $L_{s}$ is distance between receiver and background radiation source. The optical signal-to-noise ratio (OSNR) at the output of the post-amplifier is

(25) $$\mathrm{OSNR}=\frac{P_{out}}{2\rho B_{opt}},$$

where *B_opt_* = 12.5 GHz is the reference bandwidth. From Equations (13)–(15), it follows that for the given transmitter output power, the uplink has the lowest OSNR and sat-to-sat link has the highest OSNR.

## 5. Simulation Results and Discussion

We have carried out the Monte-Carlo simulations of the FSO system using the parameters listed in Table 1, unless otherwise specified. At transmitter, 6,553,600 symbols were transmitted with QAM-16 modulation format and 40 Gb/s symbol rate with the link distance 35,000 km to simulate the coherent and KK transceivers performance. The logarithm appearing in Equation (7) for the phase extraction in KK receivers leads to spectral broadening and hence, digital up-sampling of the received photocurrent is required. For the simulation of the transmitter and channel, a sampling rate of 20 GSa/s is used, and at the receiver digital signal processing (DSP), the sampling rate is increased to 40 GSa/s. Increasing the sampling rate beyond 40 GSa/s did not provide any performance improvement of the KK receiver.

There are three types of atmospheric turbulence, i.e., beam wander, beam spreading, and beam scintillation. Out of these three types, for simplicity, we neglected the beam wandering in our simulations. Beam wander is caused mainly by large-scale turbulence near the transmitter and, therefore, can be typically neglected for downlink scenarios [4,25]. For uplink, we assume that the beam is well tracked, and beam wander is mitigated [28,29,30,31]. In our simulations, we have used the Hufnagel–Valley model for turbulence [4]. The normalized variance of scintillation is

(26) $$\sigma_{I}^{2}=\frac{\langle I^{2}\rangle }{{\langle I\rangle }^{2}}-1$$

where 〈I〉 and $\langle I^{2}\rangle$ are the mean and second moment of the optical intensity at the receiver, respectively. When $\sigma_{I}^{2}\leq 1$, it is considered as the weak turbulence and when $\sigma_{I}^{2}\geq 1$, it is considered as moderate-to-strong turbulence [4]. In the presence of weak turbulence, the time-varying received optical intensity may be described by a log-normal probability density function (PDF) [4] as

(27) $$P(I)=\frac{1}{I\sqrt{2\pi \sigma_{I}^{2}}}\exp\left(\frac{-{\left[\ln\left(\frac{I}{I(0,L)}\right)\right]}^{2}+0.5\sigma_{I}^{2}}{2\sigma_{I}^{2}}\right)$$

Also, in the presence of moderate and strong turbulence, we used the extended equations presented in [4] for Gamma–Gamma distribution, specifically for the uplink. More details can be found in [4]. In the case of coherent receiver without turbulence, for M-ary QAM, the symbol error rate is given by [31]

(28) $$P_{e}=4\left(1-\frac{1}{\sqrt{M}}\right)\;Q\left(\sqrt{\frac{3\log_{2}M\rho }{M-1}}\right)\left(1-\left(1-\frac{1}{\sqrt{M}}\right)Q\left(\sqrt{\frac{3\log_{2}M\rho }{M-1}}\right)\right),$$

where ρ is the signal-to-noise ratio (SNR) per bit and Q(.) is the Q-function. In the presence of turbulence, the mean BER is calculated by

(29) $$<\mathrm{BER}>=\int_{-\infty }^{\infty }p(I)\mathrm{BER}(I)dI,$$

where p(I) is the probability density function of turbulence and I is the optical intensity.

### 5.1. Comparison of KK and Coherent Systems without Scintillation

**Figure 4:**
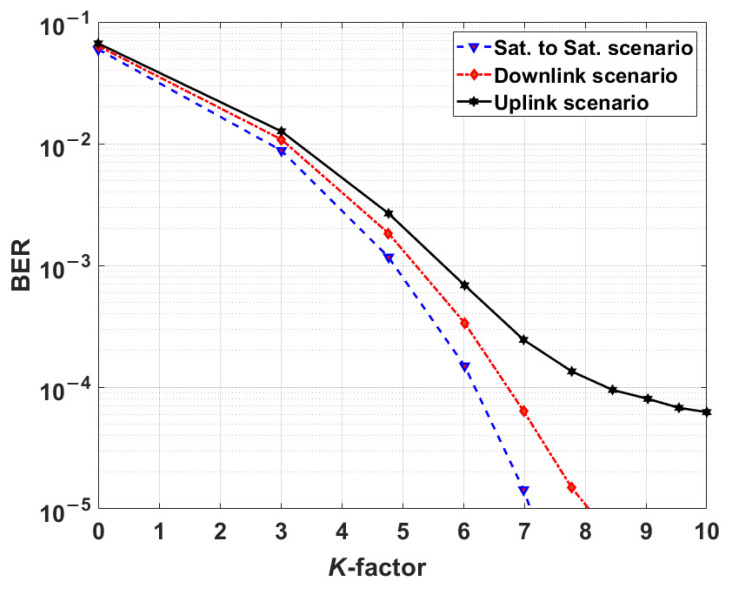
KK transceiver performance versus *K*-factor for satellite-to-satellite, downlink, and uplink communications.

In this section, we study the KK and coherent transceivers performances by neglecting turbulence (see Section 5.2 for results in the presence of turbulence). However, for uplink and downlink configurations, the path losses due to turbulence are included (see Equations (13)–(15)). Figure 4 shows the BER versus the *K*-factor (i.e., *K* of Equation (1)) for three FSO scenarios (see Figure 1) that are sat-to-sat, downlink, and uplink FSO communications. As can shown in Figure 4, the BER decreases as the *K*-factor increases for all scenarios. As the K-factor increases, the penalty due to phase extraction using the Hilbert transform decreases, and when this penalty becomes zero, we see saturation of the BER for the uplink. Since the uplink has the lowest SNR among these three schemes, saturation of the BER occurs for relatively lower *K*-factor since the channel noise dominates over the noise associated with the phase extraction. Note that this saturation effect would happen to the other links at higher K-factors because of their higher OSNRs.

**Figure 5:**
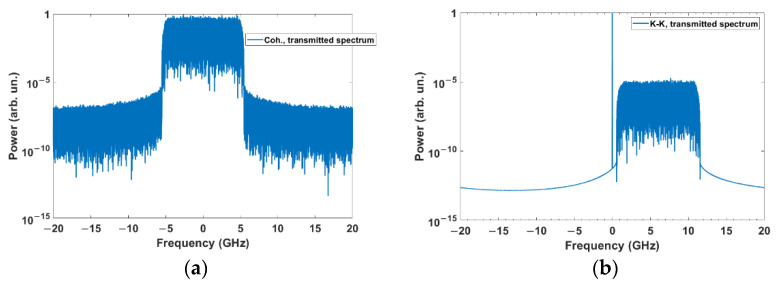
Normalized spectrum of the transmitted signal (**a**) coherent communication system, and (**b**) KK communication system (*K* = 10).

**Figure 6:**
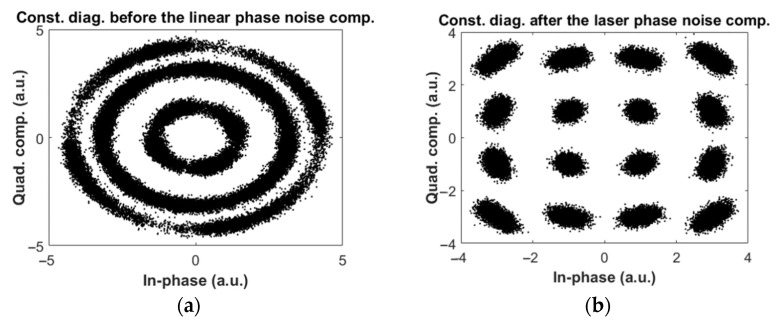
Example of received constellation diagram for QAM-16 in FSO coherent system (downlink), (**a**) before phase noise compensation, and (**b**) after phase noise compensation.

**Figure 7:**
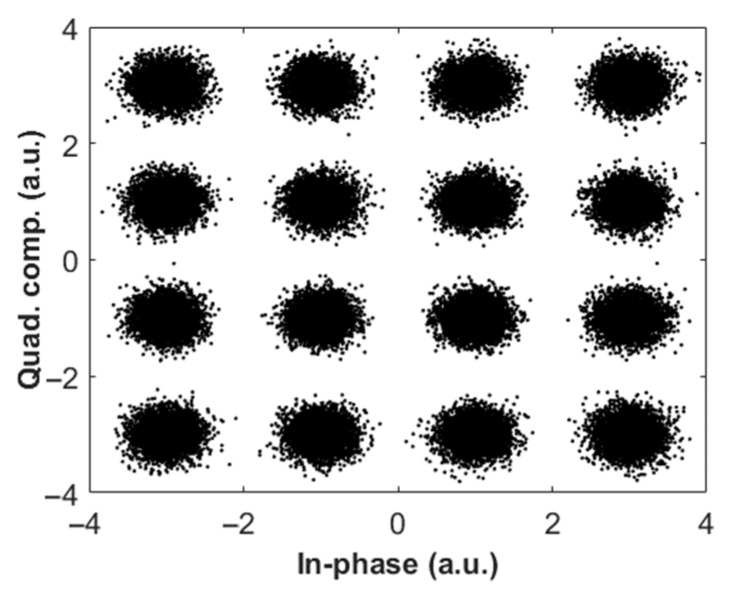
Example of received constellation diagram for QAM-16 in FSO KK system (downlink, *K* = 10).

Figure 5a,b show the transmitted spectra of the FSO system based on coherent and KK transceivers. In Figure 5b, there is a DC component (see Equation (1)) which is required to ensure the minimum phase property. An example of the received constellation diagram for coherent transceiver is shown in Figure 6 for the downlink scenario. Due to laser phase noise introduced by the transmitter and receiver (LO) lasers [32], constellations are lost (Figure 6a) which are recovered by digital phase noise compensation (Figure 6b). We have implemented the phase noise compensation technique developed in [33] for the case of a coherent transceiver. However, for the case of the KK transceiver, the received signal is not sensitive to transmitter laser phase noise because of the direct detection, and, hence, phase noise compensation is not required for this FSO system. Figure 7 shows the constellation diagram for the case of KK transceivers. Comparing Figure 6b and Figure 7, we see that the impact of noise is higher in a system based on KK transceiver (electrical SNR is the same for both systems). However, there is residual phase noise in Figure 6b at the corners even after the phase noise compensation. It is due to the fact that as the channel noise and/or constellation size increases, the compensator cannot compensate for the phase noise exactly.

**Figure 8:**
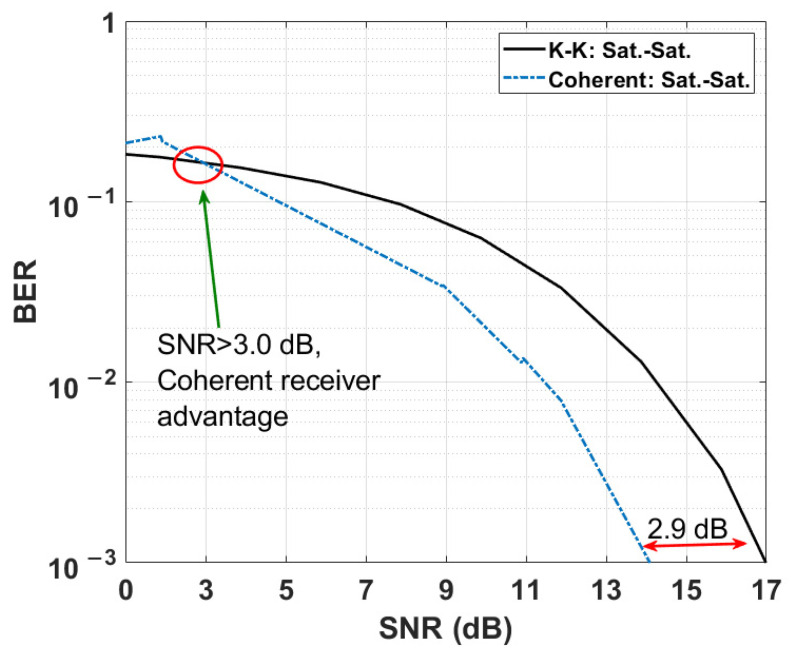
Comparison between coherent and KK transceivers performance versus electrical SNR (For KK transceiver: *K* = 10).

**Figure 9:**
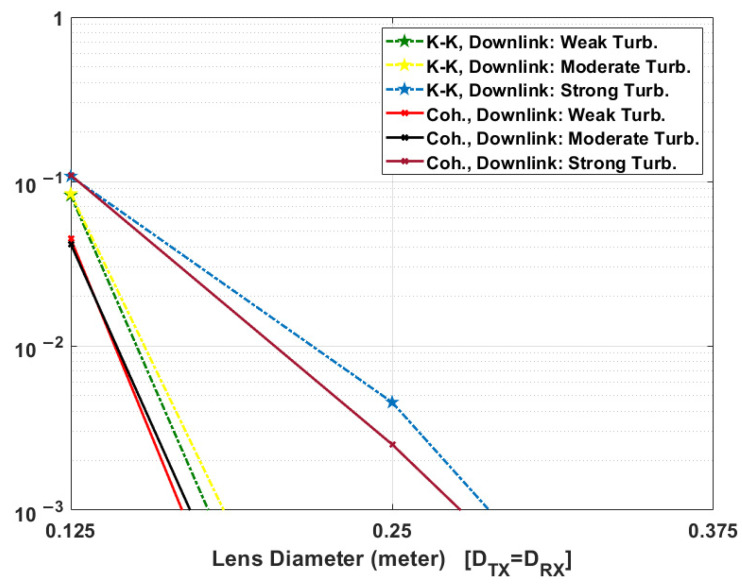
Comparison between KK and coherent transceiver downlink performance in the presence of scintillation versus telescopes lens diameter (Tx/Rx amplifier gains are 30 dB, Tx/Rx lens diameters are assumed equal. Also, please see $C_{n}^{2}\left(0\right)$ values in Table 1 for weak, moderate, and strong turbulence.

**Figure 10:**
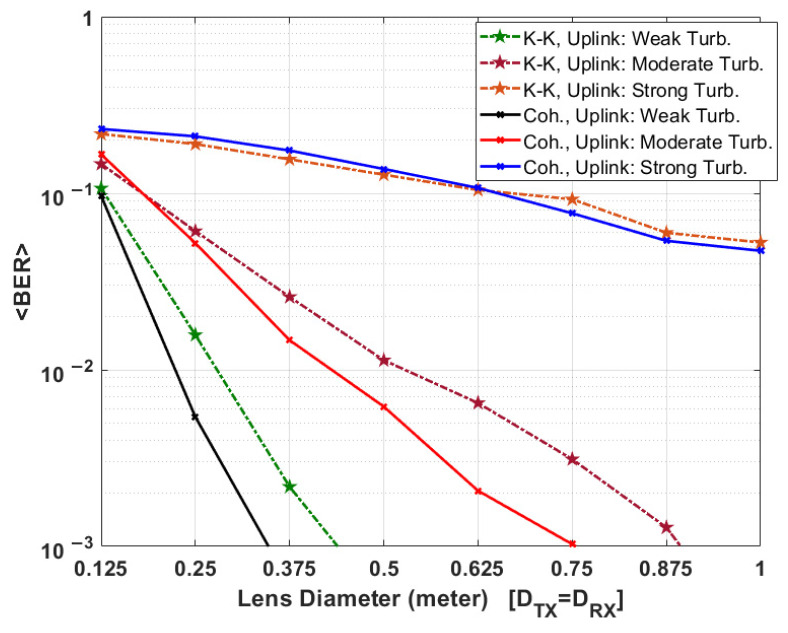
Comparison between KK and coherent transceiver uplink performance in the presence of scintillation versus telescopes lens diameter (Tx/Rx amplifier gains are 30 dB, Tx/Rx Lens diameters are assumed equal. Also, please see $C_{n}^{2}\left(0\right)$ values in Table 1 for weak, moderate, and strong turbulence).

Next, we compare the BER of KK and coherent transceivers for the sat-to-sat scenario in Figure 8. In the case of KK receiver, the electrical SNR is calculated after the matched filter (see Figure 3). As shown, the electrical SNR required to achieve a BER of 10^−3^ for KK receiver is 2.9 dB higher than that of coherent receiver. On the other hand, at a fixed SNR of 14.3 dB, the BER for KK transceiver is 1.03 × 10^−2^ (see Figure 8) and the required hard decision forward error correction (HD-FEC) overhead (OH) for BER < 1.03 × 10^−2^ is 14.3% [32,33]. However, the required OH for coherent transceiver is as low as 6% [34] at the fixed SNR of 14.3 dB, since the BER is less than 10^−3^ at this SNR. So, the 8.3% OH is the penalty for using KK system instead of coherent system, which is acceptable for reduction of the cost and complexity of FSO coherent communication system. It may be noted that this comparison is made for the laser linewidth of 22 kHz (see Table 1). If the linewidth is increased to 1 MHz, the system based on coherent receiver will incur a serious penalty (even with digital phase noise compensation) whereas the performance of the system based on KK transceiver will not be impacted. We note that the 2.9 dB SNR penalty is the price to pay for using inexpensive KK transceiver instead of coherent transceiver, although this penalty is lower at lower SNRs. In this comparison (and in Figure 9 and Figure 10 as well), the waste of transmitter power due to pilot beam in the KK scheme and the mode mixing loss in the coherent detection scheme are not considered.

### 5.2. Comparison between KK and Coherent Systems in the Presence of Scintillation

The sat-to-sat link can be assumed to be free from atmospheric turbulence. But the transceiver performance for downlink and uplink scenarios is impacted in the presence of turbulence. In this section, we compare the performance of coherent and KK transceivers for downlink and uplink scenarios in the presence of weak, moderate, and strong turbulence close to the ground station telescope. For simplicity, we assumed that the lens diameter of the Tx and Rx telescopes are equal.

To compute the mean BER in the presence of turbulence, we proceed as follows. The received optical intensity is divided into *N* blocks. Let the range of optical intensity in the *n*th block be (*I_n_*, *I*_*n*+1_). This range is so selected that the BER is approximately constant. Monte-Carlo simulation of the FSO system with a constant path loss is carried out using 65,536 symbols assuming that the optical intensity is (*I_n_* + *I*_*n*+1_)/2 and the corresponding bit error rate, *BER_n_* is computed. This procedure is repeated for all the *N* blocks and a lookup table that maps the optical intensity to BER is formed. Using the H-V turbulence model [4], let the chance of the optical intensity lying in the range (*I_n_*, *I*_*n*+1_) be *p*(*n*). The mean BER is computed as

(30) $$<\mathrm{BER}>=\sum_{n}p(n){\mathrm{BER}}_{n}$$

Figure 9 and Figure 10 show the mean BER performance of coherent and KK systems, respectively (Tx/Rx amplifier gains are 30 dB). In general, the mean BER decreases as the lens diameter increases since a large diameter leads to a lower path loss. For both downlink and uplink scenarios, in the presence of weak and moderate turbulence, the required lens diameter of telescopes in coherent transceiver is lower to achieve the same mean BER performance in comparison to KK transceiver. For example, for uplink, in the presence of weak turbulence, the required lens diameter to achieve <BER> = 10^−3^ is ~35 cm and ~44 cm for coherent and KK transceivers, respectively (see Figure 10). This translates into a SNR penalty of 2.0 dB for KK transceiver. For downlink, in the presence of strong turbulence, the required lens diameter to achieve <BER> = 10^−3^ is ~27.5 cm and ~28.8 cm for coherent and KK transceivers, respectively (see Figure 9). This translates into a SNR 0.4 dB penalty for KK transceiver. It may be noted that the SNR penalty at a BER of 10^−3^ for KK receiver is 2.9 dB (see Figure 8) in the absence of turbulence which is much larger than 0.4 dB in the presence of strong turbulence. In other words, the coherent receiver loses one of its main advantages in the presence of strong turbulence. In Figure 10, in the presence of moderate and strong turbulence, for the lens diameter less than 62.5 cm (20 cm) for strong (moderate) turbulence, KK transceiver performs slightly better than the coherent transceiver. The reason is that in the presence of strong/moderate turbulence, path loss is so high for uplink that the SNR is very low and the performance of laser phase noise compensator decreases at lower SNR for coherent receiver (it happens in Figure 8 too) whereas the phase noise is not an issue for KK receiver.

Due to turbulence, the optical intensity at the receiver is time variant, which leads to a time-varying SNR. Therefore, at a weak received optical intensity level, the SNR decreases significantly resulting in the BER degradation. For example, in the uplink scenario, for *K* = 10 and lens dimeter of 0.25 m at transmitter and receiver, the BER of $6.8\times 10^{-5}$ in the absence of turbulence shown in Figure 4, increased to the mean BER of $2\times 10^{-2}$ in the presence of turbulence (see Figure 10). Therefore, turbulence limits the information rate of FSO communications. Also, to make FSO link more reliable in the presence of strong turbulence in both KK and coherent transceivers, FEC codecs such as low-density parity-check (LDPC), or polar codes are required [35,36,37].

In summary, in Table 2, we show the qualitative comparison between KK, coherent and intensity-modulated direct-detection (IMDD) systems. Here, the cost of a KK system is lower than that of a coherent system since expensive ECL and LO laser are not required for the KK system. In KK system, Hilbert transform is implemented in digital domain to extract the signal phase and hence, the computational cost of the KK system is higher than that of the IMDD system. The mode mixing efficiency is acceptable in KK and IMDD, typically more than 80%. However, for the coherent detection in FSO communication systems in the presence of atmospheric turbulence, the data-LO mode mixing efficiency is poor [20,21,22]. The KK system enables the simultaneous amplitude and phase modulation similar to coherent detection at low hardware cost and yet, it does not suffer from poor mode mixing efficiency in the presence of atmospheric turbulence.

## 6. Conclusions

We have proposed and numerically implemented a free space optical (FSO) communication scheme based on KK transceivers. Numerical simulations of ground-to-satellite (uplink) and satellite-to-ground (downlink) in the presence of weak, moderate, and strong turbulences were carried out. Our results show that the KK transceivers provide transmission performance comparable to coherent receivers, but at a significantly lower system cost and complexity. KK receiver uses a direct detection receiver and extracts the signal phase using Hilbert transform in the digital domain. Unlike the coherent receiver, KK receiver does not require a laser at the receiver. In addition, KK receiver is not sensitive to the transmitter laser phase noise and hence, conventional distributed feedback (DFB) lasers with relatively large linewidths can be used at the transmitter. In contrast, for coherent detection, expensive external cavity lasers with very small linewidths (<100 kHz) should be used. This smaller linewidth criterion becomes even more critical as the size of QAM constellation increases. KK transceiver requires only a single Mach–Zehnder modulator whereas the coherent transceiver requires a more complex optical I-Q modulator. Numerical simulation of 40 Gb/s, 35,000 km sat-to-sat link with 16-QAM showed that there is about 2.9 dB SNR penalty at a BER of 10^−3^ for KK transceivers as compared to coherent receivers. However, in the presence of strong turbulence, the SNR penalty at a BER of 10^−3^ for KK receivers becomes quite small (~0.4 dB).

## Figures and Tables

**Table 1:** FSO KK and coherent system simulation parameters.

Simulation Parameters	Value/Variable
Pre-amp. gain (Tx)	30 (dB)
Post-amp. gain (Rx)	30 (dB)
Noise figure (NF) pre-amp (Tx)	5
Noise figure (NF) post-amp (Rx)	3.3
Average transmit signal power (PT)	30 (dBm)
Number transmitted symbols	6,553,600
Modulation format	16 QAM
Telescope diameter (Tx)	0.25 (m)
Telescope diameter (Rx)	0.25 (m)
Data rate	40 (Gb/s)
Over-sampling factor	4
Symbol rate	10 (GS/s)
Pulse shape	Root-raised cosine
Roll-off factor	0.1
Link distance	35,000 (km)
Zenith angle	0 (deg)
PD responsivity	0.8 (A/W)
Laser wavelength	1550 (nm)
RMS of wind speed	21 (m/s)
Wind speed close to ground	5 (m/s)
Tropopause height	9.4 (km)
Tropopause thickness	4.8 (km)
Turbulence model [4]	Hufnagel-Valley model (H-V model)
Structure parameter at height h using H-V model [4]	$C_{n}^{2}\left(h\right)$
$C_{n}^{2}\left(0\right)$ Turbulence close to ground (weak)	1.7 × 10^−14^ (m^−2^/3) [4]
$C_{n}^{2}\left(0\right)$ Turbulence close to ground (moderate)	1.0 × 10^−13^ (m^−2^/3) [4]
$C_{n}^{2}\left(0\right)$ Turbulence close to ground (strong)	2.0 × 10^−11^ (m^−2^/3) [27]
Divergence factor (Tx)	0.942
$\mathrm{Misalignment}\;\mathrm{pointing},\;L_{P}$	0.2
$\mathrm{Tx}\;\mathrm{efficiency},\;\eta_{T}$	0.8
$\mathrm{Rx}\;\mathrm{efficiency},\;\eta_{R}$	0.8
$\mathrm{Rx}\;\mathrm{telescope}\;\mathrm{transmission}\;\mathrm{factor},\;T_{R}$	0.8
$\mathrm{Tx}\;\mathrm{telescope}\;\mathrm{transmission}\;\mathrm{factor},\;T_{T}$	0.8
Responsivity R,	1.1 (A/W)
Atmospheric loss (downlink/uplink), A_atm_	1.0 (dB)
Fried parameter, r0 (weak turbulence)	200 (mm)
Fried parameter, r0 (moderate turbulence)	80 (mm)
Fried parameter, r0 (strong turbulence)	20 (mm)
Background noise (BN) (daytime, max sunlight, clear sky)	1.544 × 10^−25^ (W/Hz)
Optical filter bandwidth	12.5 (GHz)
Absolute temperature	290 (°K)
Laser linewidth (Tx)	22 (KHz)

**Table 2:** Comparison between KK, coherent and IMDD.

Performance Factor	KK	Coherent	IMDD
Transmitter power efficiency	Low	High	Low
Hardware cost [38]	Low	High	Low
Mode mixing efficiency	Good	Low	Good
DSP computational cost	Moderate	High	Low

## Data Availability

Not applicable.
